# Influence of mhealth interventions on gender relations in developing countries: a systematic literature review

**DOI:** 10.1186/1475-9276-12-85

**Published:** 2013-10-16

**Authors:** Larissa Jennings, Laina Gagliardi

**Affiliations:** 1 Department of International Health, Johns Hopkins Bloomberg School of Public Health, Baltimore, MD, USA; 2 Department of Population, Family, and Reproductive Health, Johns Hopkins Bloomberg School of Public Health, Baltimore, MD, USA

**Keywords:** mHealth, Mobile phone technology, Gender relations, Equity, Access, Women, Developing countries, Literature review

## Abstract

**Introduction:**

Research has shown that mHealth initiatives, or health programs enhanced by mobile phone technologies, can foster women’s empowerment. Yet, there is growing concern that mobile-based programs geared towards women may exacerbate gender inequalities.

**Methods:**

A systematic literature review was conducted to examine the empirical evidence of changes in men and women’s interactions as a result of mHealth interventions. To be eligible, studies had to have been published in English from 2002 to 2012, conducted in a developing country, included an evaluation of a mobile health intervention, and presented findings on resultant dynamics between women and men. The search strategy comprised four electronic bibliographic databases in addition to a manual review of the reference lists of relevant articles and a review of organizational websites and journals with recent mHealth publications. The methodological rigor of selected studies was appraised by two independent reviewers who also abstracted data on the study’s characteristics. Iterative thematic analyses were used to synthesize findings relating to gender-transformative and non-transformative experiences.

**Results:**

Out of the 173 articles retrieved for review, seven articles met the inclusion criteria and were retained in the final analysis. Most mHealth interventions were SMS-based and conducted in sub-Saharan Africa on topics relating to HIV/AIDS, sexual and reproductive health, health-based microenterprise, and non-communicable diseases. Several methodological limitations were identified among eligible quantitative and qualitative studies. The current literature suggests that mobile phone programs can influence gender relations in meaningfully positive ways by providing new modes for couple’s health communication and cooperation and by enabling greater male participation in health areas typically targeted towards women. MHealth initiatives also increased women’s decision-making, social status, and access to health resources. However, programmatic experiences by design may inadvertently reinforce the digital divide, and perpetuate existing gender-based power imbalances. Domestic disputes and lack of spousal approval additionally hampered women’s participation.

**Conclusion:**

Efforts to scale-up health interventions enhanced by mobile technologies should consider the implementation and evaluation imperative of ensuring that mHealth programs transform rather than reinforce gender inequalities. The evidence base on the effect of mHealth interventions on gender relations is weak, and rigorous research is urgently needed.

## Introduction

The gender divide in access to and use of mobile phone technologies is well known [1-5]. Strategies around the globe have aimed to improve women’s eligibility to participate in mHealth initiatives, or health programs enhanced by mobile phone technologies, by targeting women’s barriers to own and use mobile phones [1,2].

However, despite evidence on the positive effects of mHealth interventions on women’s health and care-seeking, there is growing concern that mobile-based programs geared towards women may exacerbate gender inequalities. Particularly in settings where men have historically limited women’s autonomy and decision-making, little is known regarding the full impact of mHealth programs on the relational experiences of men and women [6,7].

Evidence suggests that while mHealth programs hold the potential to shift gender roles by empowering women through improvements in knowledge, decision-making, and economic gain [8,9], some mHealth interventions may exacerbate gender inequalities by reinforcing existing power differentials [10-12]. For example, mHealth projects which target female mobile phone owners or provide mobile phones to women may have harmful consequences within conjugal relationships brought on by women’s mobile-enhanced autonomy and decision-making ability [1,13]. Such changes may increase women’s risk of domestic violence and privacy invasion, in addition to increasing men’s monitoring of women’s whereabouts and communication. Shifts in household spending due to increased mobile airtime expenses may also aggravate existing household dynamics.

The term, gender relations, refers to “varying roles and relations between women and men which are influenced by socio-cultural, political, economic, religious, and environmental factors” [13]. Recent discourse has highlighted a growing need to develop “gender-transformative” initiatives that promote relational equality rather than implement programs which accommodate or ignore gender imbalances by doing little to address them [14]. There are concerns likewise regarding the implementation of “gender-exploitative” programs which inadvertently rely on power differences to achieve intervention goals [14]. Yet, despite the need to develop appropriately empowering and safe mobile health programs for women, there has been no systematic review to-date to examine what is currently known on the effect mHealth initiatives on gender relations in developing countries [10,15]. While understanding the technology-gender relationship has been a growing area of research [11,13,16], many studies have focused on what Silverstone, Hirsch, and Morley (1992) refer to as *appropriation*, or gendered access and ownership of technological resources, with less attention to *incorporation* or the influences of technology on gender power relations [17]. This represents an important research gap regarding the programmatic effects on women’s relational experiences, especially in settings where women must overcome social, financial, and device literacy barriers, including spousal disapproval, in order to take part in mHealth interventions [1,2,10,18]. This review aimed to synthesize the empirical evidence of changes in men and women’s interactions as a result of participation in mHealth interventions [15,16].

## Methods

### Inclusion criteria

Research studies that met the following criteria were included: (i) the study evaluated a mobile phone health intervention or intervention aiming to improve women’s mobile phone ownership and use; (ii) the study was conducted in a developing country, defined on the basis of the World Bank categories for low-income, lower-middle income, or upper-middle income economies [19]; (iii) outcomes or observations relating to gender relations were reported; and (iv) the study was published in English between January 2002 and December 2012. Peer-reviewed and gray literature evaluations of all types, including qualitative, formative, or process evaluations were eligible. Exclusion criteria included non-English-language studies, studies conducted in developed countries, unpublished reports (such as dissertations or conference abstracts), non-intervention studies, mHealth interventions targeting health workers, or studies where mobile phones were used for data collection rather than intervention purposes.

### Search strategy

A preliminary literature search was conducted to identify relevant search terms and guide the development of study appraisal documents. The search strategy included an electronic and manual search. The electronic search was the primary means of identifying relevant studies and comprised of four electronic bibliographic databases of peer-reviewed literature: MEDLINE/PubMed, PsycINFO, SciVerse Scopus, and Excerpta Medica Database (EMBASE). These databases were selected to cover a broad range of disciplines, including medical and social science research. The search terms included Boolean-paired key word sets of synonyms and spelling variations (where applicable) relating to mobile phones, maternal and child health-related interventions, and gender relations [Table 1].

**Table 1:** Search strategy for electronic databases

**MEDLINE/PubMed, EMBASE, Scopus, PsycINFO**					
**Search category**	**Mobile phones**		**Maternal and child health-related interventions**		**Gender relations**
**Search terms**	mobile phone(s); cellular phone(s); cell phone(s); mobile; phone(s); mobile-based; SMS; text(s); text-message(s); audio message(s); smart phone(s); mobile health; mHealth; eHealth	**AND**	health; maternal; birth(s); child(ren); delivery; obstetric; neonatal; pregnancy; anemia; preeclampsia; HIV; AIDS; malaria; antenatal; abortion; tuberculosis; postpartum; family planning; sexual; sex; reproductive	**AND**	gender; sex; women; female; relations; interaction(s); equity; inequity; equality; inequality; men; male; participation; empower(ment); (wo)men’s role(s); autonomy; violence; safety; literacy; economic; mobility; status; access; capacity; communication(s)

To obtain non-peer reviewed or gray literature, a manual review of nine websites of key organizations within the mHealth area was conducted: GSMA mWomen, Mobile Active, mHealth Alliance, Knowledge for Health, International Center for Research on Women, Women for Women International, Mobiles for Education Alliance, mHealth Info, and Health Unbound. The manual search also included a review of articles published from 2010 to 2012 in eight priority journals relating to mHealth or among which mHealth-related articles had recently been published. These included: Telematics and Informatics; Journal of Telemedicine and Telecare; Technology and Health Care; Gender; Technology and Development; BMC Pregnancy and Childbirth; Reproductive Health; AIDS and Behavior; and Contraception. The tables of content prior to 2010 of the priority journals were not reviewed given the relatively recent implementation of mHealth interventions and based on the limited number of eligible studies published before 2010 as identified in the electronic search.

### Title, abstract, and article screening

**Figure 1:**
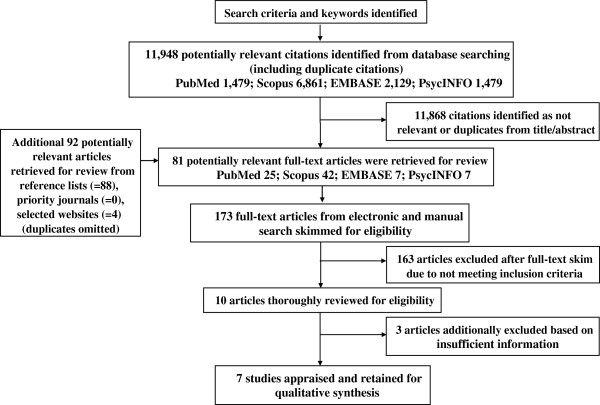
Search and Screening Flow Chart.

Figure 1 illustrates the search and screening process. Once the search terms were applied to the electronic databases, an initial selection of potentially relevant citations was based on the screening of the title and abstract by the primary reviewer. Given that the title and abstract did not provide sufficient information to determine eligibility, the full-text publications of all potentially relevant citations were acquired and skimmed by two reviewers who independently excluded articles not meeting the inclusion criteria. Full-text publications were also retrieved and skimmed for eligibility from reference lists of relevant articles, priority journals, and selected websites. All citations were entered into a database to track the retrieval process. Discrepancies in study eligibility from the initial skim were discussed and corrected based on consensus between the two reviewers. A second, more thorough reading of selected studies was used to determine, based on consensus, the final set of studies retained for the review.

### Study appraisal

All final selected studies underwent a systematic appraisal by the two reviewers [Table 2]. Data were extracted and documented in a study appraisal form for the following items: article identification number, author, year of publication, search strategy source, publication journal (if applicable), country, study design, mHealth intervention description, study objective, sample/participants, data collection method, theoretical framework, primary outcome(s), gender-relations related indicator(s), and key gender-relations related findings. All corresponding completed appraisal forms were compared. Discrepancies were resolved in discussion prior to being added to the final appraisal database.

**Table 2:** Characteristics of selected studies

**Author(s) Journal**	**[ *Health area *] MHealth intervention**	**Country**	**Study’s primary objective**	**Design**	**Sample**	**Findings on gender relations**
**(1) Akinfaderin-Agarau *et al.*** [35]	*Sexual Health and HIV/AIDS*:	Nigeria	To determine reasons for significantly low uptake among young women	Single group, posttest design using mixed methods	726 users	*Non-Transformative:* Participation in mHealth intervention hindered by gender-based expectations of women, including fear of spousal distrust and invasions of privacy.
	Users submitted questions via SMS and received accurate and confidential sexual health and HIV/AIDS information					
African Journal of Reproductive Health						
**(2) Balasubramanian *et al.*** [36]	*Microenterprise*: Illiterate and semi-literate women received daily mobile phone audio messages on business strategies for a goat-rearing enterprise, along with maternal and child health information, linked with reduced network call-rates	India	To examine effects of mobile phones as a tool for enterprise and learning on women’s social standing	Single group, posttest design using mixed methods	73 program participants	*Transformative and Non-Transformative:* Ownership of new technology raises status of women within household, but creates some tensions regarding use. In some cases, perpetuated male hierarchies and dependence.
Distance Education						
**(3) Chib *et al.*** [37]	*HIV/AIDS*:	Uganda	To assess mobile participation rates and HIV/AIDS-related knowledge	Single group, posttest design using mobile quiz response data	2,363 mobile quiz takers	*Non-Transformative:* Two-fold higher response rates among men than women. Authors consider technology use reflected and further entrenched gender inequities.
	Users enhanced HIV/AIDS knowledge and awareness by answering SMS-based multiple choice quizzes with free HIV counseling and testing referrals and related prizes					
Journal of Health Communication: International Perspectives						
**(4) Corker**[38]	*Reproductive Health:*	Congo	To determine hotline intervention’s reach and participation among men and women	Single group, posttest design using call data	20,036 calls	*Transformative and Non-Transformative:* New means of engaging men and potentially increasing partner communication, but reflected and reinforced existing power differentials and mobile divide.
Cases in Public Health Communication & Marketing	Couples used toll-free hotline to obtain confidential family planning information and referrals to family planning clinics and pharmacies					
**(5) Misraghosh *et al.*** [40]	*Microenterprise:*	India	To examine effects of mobile retail business on women’s empowerment	Single group, pre-/posttest design using mixed methods	50 women mobile distributors	*Transformative (Positive/Negative):* Strengthened women’s earning and decision-making power. Also shifted men's role as predominate breadwinner, resulting in some tensions and abuse.
	Women entrepreneurs sold and distributed mobile phone products to other women with health-added services and mobile phone literacy training					
GSMA mWomen Programme						
**(6) L’Engle *et al.*** [39]	*Reproductive Health:*	Tanzania	To evaluate mHealth intervention’s feasibility, reach, and effect on contraception use	Single group, posttest design using mobile queries and questionnaire	1,142 phone query users	*Transformative:*
	Users opted-into an interactive and menu-based SMS portal that provided automated information about family planning methods.					Created new channel for male engagement and potential increased couple’s communication on family planning.
Contraception						
**(7) Odigie *et al.*** [41]	*Cancer*:	Nigeria	To determine platform effectiveness to improve communication and patient follow-up rates	Single group, posttest design using structured in-person interviews	1,160 oncology patients	*Transformative and Non-Transformative:* New communication mode for women circumvents spousal dependence and permission. Yet, men make calls on behalf of women to sustain decision-making roles.
	Patients received SMS cancer treatment reminders and were invited to utilize a call-hotline to talk to registered physicians/health providers about cancer-related health concerns and psycho-social support.					
Psycho-Oncology						

### Quality assessment

As part of the study appraisal, the methodological rigor was assessed for each article using an 11-item quality assessment checklist for quantitative studies and a 10-item quality assessment checklist for qualitative studies [Table 3]. Mixed methods studies were assessed using quantitative and qualitative checklists. Inclusion of items for the quality assessments was informed by published guidelines and indexes for examining the quality of quantitative [20-24] and qualitative studies [24-27]. The checklist for quantitative studies used 11 items to evaluate the study’s methodological quality: longitudinal/prospective design; pre-post measure of the outcome(s) of interest; use of control or comparison group; comparison group selected from a similar population with regard to pre-intervention outcomes or socio-demographics; justified sample size; random assignment of individuals to the intervention group; outcome(s) of interest measured objectively; response or follow-up rate of more than 80%; use of theoretical framework; report of an index of variability (such as an estimate of variance, confidence interval, or other test statistic); and report of program implementation detail sufficient for replication. The 11 items were scored as 0 (=not found) or 1 (=found) and summed for a total of 11 points possible. Based on the total quality score, each study was categorized within one of three ratings: weak rating (less than or equal to 5 points), moderate rating (6 to 8 points), and strong rating (9 to 11 points).

**Table 3:** Summary of quantitative and qualitative quality scores for selected articles

**Quantitative studies (N= 6)**	**Number of articles**	**% Total studies**
1 – Longitudinal/prospective design	5	83.3
2 – Pre-post measure of outcome(s) of interest	2	33.3
3 – Use of control or comparison group	1	16.7
4 – Comparison group selected from similar population with regard to pre-intervention outcomes or socio-demographics	1	16.7
5 – Sample size justified	2	33.3
6 – Random assignment of individuals to intervention	0	0
7 – Outcome of interest measured objectively and systematically	6	100
8 – Response or follow-up rate of more than 80%	3	50
9 – Use of theoretical framework for guidance	1	16.7
10 – Report of an index of variability between groups	1	16.7
11 – Report of intervention implementation detail to facilitate replication	6	100
*Strong Rating* (9 – 11 points)	0	0
*Moderate Rating* (6 – 8 points)	2	33.3
*Weak Rating* (≤ 5 points)	4	66.7
**Qualitative studies (N= 3)**	**Number of articles**	**% Total studies**
1 – Prolonged engagement in study setting	2	66.7
2 – Justification for design and methods selected	3	100
3 – Sampling strategy justified	1	33.3
4 – Analytical methods clearly described	1	33.3
5 – Use of verification methods to demonstrate credibility	1	33.3
6 – Reflexivity of account provided	0	0
7 – Detailed report of findings	3	100
8 – Balanced and fair representation of view points	2	66.7
9 – Conclusions supported and confirmable by the data	3	100
10 – Report of intervention implementation detail to facilitate replication	2	66.7
*Strong Rating* (8 – 10 points)	0	0
*Moderate Rating* (5 – 7 points)	3	100
*Weak Rating* (≤ 4 points)	0	0

The checklist for qualitative studies used 10 items to evaluate the study’s methodological quality. The rigor items used were: prolonged engagement in study setting; justification for design and methods selected; sampling strategy justified; analytical methods clearly described; use of verification methods; reflexivity of account provided; detailed report of findings; balanced and fair representation; conclusions supported and confirmed by the data; and report of intervention implementation detail sufficient for replication. The 10 items were scored similarly as the quantitative index with less than or equal to 4 points representing a weak rating, 5 to 7 points representing a moderate rating, and 8 to 10 points representing a strong rating. For mixed methods studies, separate quality ratings were calculated for the qualitative and quantitative methods presented in the article. Non-applicable assignments were not allowed for items in either of the quality indexes. Similar to the appraisal process, the quality scores were determined independently by both reviewers and then compared. Score discrepancies were resolved through discussion. In most cases, the reviewers agreed on study appraisal and quality assessment determinations.

### Synthesis process

Findings and implications related to the influence of mHealth interventions on gender relations were synthesized using a thematic approach. This is commonly used to summarize themes identified in systematic literature reviews of qualitative and quantitative studies [28-30]. Similar to the analysis of textual data, the process of synthesizing findings among selected articles comprised an initial round of article memoing in which the reviewers independently documented analytical interpretations of findings to capture emerging themes [31,32]. Each article was then read several times to extract and group related results. We present findings related to two themes identified from the synthesis process and as discussed in the growing body of literature on mHealth and gender. The first theme relates to *transformative influences* on gender relations, representing positive relational changes which empower women as well as negative relational changes brought by gender-based tensions [11,13,16,33]. The second theme relates to *non-transformative influences* on gender relations, representing ways in which participation in mobile health interventions perpetuate rather than challenge gendered-based inequalities [11,12,16,34].

## Results

### Literature search and review process

A total 173 full-text publications were retrieved for review from 11,948 potentially relevant citations based on the publication’s title and abstract (Figure 1). The retrieved full-text articles were skimmed for eligibility, and 163 were excluded. The most common reasons for exclusion were absence of an evaluation of a mHealth intervention, study location in a developed country, or absence of reported findings on gender relations. Following a full and thorough screening, 3 papers were additionally excluded due to insufficient information.

### Characteristics of included studies

Seven studies were retained in the final group of articles: Akinfaderin-Agarau *et al.*[35], Balasubramanian *et al.*[36], Chib *et al.*[37], Corker [38], L’Engle *et al.*[39], Misraghosh *et al.*[40], and Odigie *et al.*[41]. The majority of selected articles were pulled from the electronic database search with the exception of two which were identified from the manual search of reference lists and related websites. Six studies were drawn from peer-reviewed journals and one was identified from the gray literature. The characteristics of the final set of articles are presented in Table 2. Although the search examined articles from 2002 to 2012, all seven were published between 2010 and 2012. Five studies were conducted in sub-Saharan Africa and two in South Asia. Several health areas were targeted using mobile phone programs, including HIV/AIDS, sexual and reproductive health, health-linked microenterprise, and non-communicable diseases. The majority of mHealth interventions consisted of SMS-based programs using interactive, quiz, and question-and-answer formats. Others were call-based interventions where participants received audio messages, accessed a hotline, or were advised to call physicians for medical counseling. A final intervention utilized female mobile phone retailers to increase women’s mobile phone uptake and literacy in addition to providing mobile-based health information. All mHealth interventions were evaluated using a single group pre-and-post or posttest only design.

### Quality of the evidence

Table 3 summarizes the quality assessment of the selected articles. Four articles used quantitative research methods, one used qualitative research methods, and two used mixed methods. Among the six studies using quantitative research methods (n=4 quantitative only and n=2 mixed methods), two were rated as moderate (6 to 8 points) and four were rated as weak (≤ 5 points). Among the three studies using qualitative research methods (n=1 qualitative only and n=2 mixed methods), all three received moderate ratings (5 to 7 points). None of the studies were judged to be of strong methodological quality. For studies with weak ratings, we did not exclude them on the sole basis of methodological rigor given the dearth of evidence on the influence of mHealth interventions on gender relations, and the need to summarize preliminary findings. This is often recommended for low-rated studies with no critical measurement or analytical deficiencies [42].

The most common strengths of the quantitative studies were longitudinal follow-up of users over time with high response rates and descriptive intervention detail. Common strengths of qualitative studies were justification for selected methods and confirmable findings based on detailed presentations of narratives and quotes. Common weaknesses of qualitative studies related to information on how study participants were sampled and how narrative data were analyzed and verified. A comparison group, randomization, power calculations, or index of variability was often omitted in quantitative studies.

### Measuring influence on gender relations

Two of the selected studies included gender relations from the outset as part of the evaluation aim. For the remaining five articles, findings relating to gender relations emerged over the course of the program or during the evaluation. Methods to examine influences on gender relations included focus group discussions, surveys, case studies, and secondary analyses of mobile phone queries and quiz responses. With the exception of mobile usage analyses, all studies examined influences of the mHealth interventions on gender relations through direct reports from women. No assessments included direct perspectives from male spousal or sexual partners. The majority of studies reported short-term findings relating to gender dynamics within mHealth interventions, rather than examining changes over time.

### Transformative influences on gender relations

The current mHealth literature described several positive transformations in the interaction of men and women. One finding was that the provision of mobile-based health information empowered couples to discuss health matters that were traditionally addressed in women-only clinical settings. Using post-intervention mobile queries, L’Engle *et al.*[39] suggested that a free and anonymous interactive short message service (SMS) portal in Tanzania created a new channel for men to access information on family planning methods in ways which did not require lengthy waits and fees at the health facility. The portal provided information on a range of family planning methods, including side effects, method effectiveness, and duration of use. Men represented almost half of the users (44%) and queried about the same number of contraceptive measures with 2.5 versus 2.1 queries compared to women, respectively [39]. L’Engle and colleagues reported that men investigated contraceptive methods for themselves as well as their female partners, suggesting greater communication among couples. Using call data from the Democratic Republic of Congo, Corker [38] also attributed men’s calling into a family planning hotline on behalf of their partners as being a positive change in gender norms in a setting where men are less likely than women to seek out health care. The authors concluded that the intervention provided a new means for men to inquire about family planning and circumvented gender norms which limited their attendance at health facilities. Such active information-gathering by men was thought to reflect increases in health-related cooperation among couples.

The literature also showed that mHealth interventions can increase women’s autonomy in seeking health information and services. Using post-intervention interviews, Odigie *et al.*[41] noted that a mobile-based physician dial-in service for women cancer patients in Nigeria increased women’s ability to directly access health care providers without relying on spousal permission or financial support for travel. The authors viewed this as dramatically altering women’s traditional lines of communication with health providers in ways that were independent from spousal control. However, the study did not examine resultant tensions, if any, among couples or the extent to which women relied on male partners for use of phones.

A third finding suggests that mHealth projects that supply women with mobile products, skills, or information valued by men can shift control of household resources and elevate the status of women. In rural India, Balasubramanian *et al.*[36] found that registering program-distributed mobile phones in women’s names meant women were more likely to be recognized by households as the phone’s owner [36]. Women entrepreneurs who were enrolled in the intervention received mobile audio messages across a range of health topics in addition to business-related information on goat-rearing. In focus groups, women reported that male household members would seek permission to use the phone, a dramatic shift from prior gender norms. Balasubramanian and colleagues also noted that women’s increased knowledge on goat-rearing from mobile audio messages raised their importance as a member of the household [36]. Also in India, to reduce the mobile phone gender gap, Misraghosh *et al.*[40] found among qualitative case studies coupled with sales data that engaging women to sell and distribute mobile phone products with health-related added services led to increased social empowerment and earning capacity. Some women reported positive responses from male spouses who also joined the mobile retail business, shifting prior male roles as the sole breadwinner for the household [40].

### Non-transformative influences on gender relations

Other findings demonstrated the potential of mHealth interventions to aggravate existing household dynamics. Balasubramanian *et al.*[36] found that in some cases gender hierarchies required rural Indian women to render phones provided by the program to their male spouse if he did not already own a phone. In these settings, women’s ownership and use of the phone was viewed unfavorably by spouses who traditionally controlled household resources. Further conflicts between couples arose as well in negotiating the use of project phones for women’s intervention purposes (such as enterprise and health information) versus communication purposes for men. Cases studies conducted by Misraghosh *et al.*[40] additionally described reports of spousal abuse by some women mobile phone retailers whose participation in the intervention challenged expectations of appropriate activities for women. Spousal demands for control and lack of spousal support resulted in some women abandoning the mobile program altogether. The authors noted that this limited their ability to take full advantage of the income generation and mobile-added health and financial services.

The evidence pointed likewise to mHealth program effects which reinforced prior relational practices, including women’s dependence on men for approval, technical, or financial support. In Nigeria, Odigie *et al.*[41] found that while an SMS cancer-treatment reminder and hotline intervention enabled women to rely less on spousal permission and financial support for care-seeking, the women’s husbands often assumed the role of speaking with the physician and arranging follow-up visits. Interviews with women attributed this to men’s role as the primary decision-maker of the household [41]. In some cases as well, Balasubramanian *et al.*[36] noted that participation in the intervention required phone literacy which the program implementers addressed during group trainings on how to use a mobile phone. However, survey data showed that nearly half (42%) of the selected women stated that they had to seek help from their spouse as a result of device and textual literacy barriers [36].

Although not a direct measure of influences on gender relations, several articles also discussed the unexpected finding relating to men’s predominant uptake of mHealth interventions which were targeted towards women. Chib *et al.*[37] and Corker [38] noted that men were likely better able to hear about, access, and pay for mobile intervention components, reflecting current gender disparities in access to mobile technologies. In Uganda, the introduction of an SMS-based HIV/AIDS campaign providing interactive quizzes and rewards resulted in a two-fold higher response rate among men as compared to women based on post-intervention mobile quiz data [37]. And, in the Congo, over 80% of callers into a family planning voice hotline were men, although the intervention’s primary target group was women of reproductive age [38]. The interventions, while successful in reaching men, were interpreted as entrenching rather than transforming gender-based inequities despite potential positive shifts in couples’ interactions.

In addition, expectations regarding the appropriate behavior of women shaped how and whether women participated in the mHealth project. In this sense, the literature suggested that mobile initiatives did little to impact gender relations, but reflected them instead. In Nigeria, Akinfaderin-Agarau *et al.*[35] attributed gendered expectations of women that discouraged their being “inquisitive” and seeking out sexual health information as a key reason for the limited enrollment in the HIV/AIDS SMS portal, which was accessed significantly more often by men [35]. In focus groups, women also stated that privacy and confidentiality concerns relating to the platform’s request for location, age, and address were reasons for non-participation. Furthermore, in households where men closely managed communication, lack of spousal permission or increased suspicion was also commonly mentioned. Given such barriers to mHealth program involvement, the authors concluded that use of mobile technologies for delivering health information in some settings resulted largely in reinforcing inequitable power relations.

## Discussion

To our knowledge, this review is the first to-date to examine the current evidence on the effect of women’s participation in mHealth interventions on gender relations in developing countries. Findings showed that while the gender disparities in access to and use of mobile technologies are well known, the rapidly growing literature on mHealth interventions provides little information on the extent to which such efforts have positively or negatively impacted gender relations.

However, despite the limited available evidence, several key findings emerged. The current literature suggests that mobile phone programs can influence the interactions of men and women in meaningfully positive ways by providing new modes for couple’s health communication and cooperation and by enabling greater male participation in health areas typically targeted towards women. The review found as well that mobile health programs can increase women’s autonomy in seeking health information and services, and when coupled with economic-strengthening activities can also elevate women’s status and resource control. We did not find strong evidence of specific adverse events such as domestic violence, mobile stalking, privacy invasions, or distrust by male partners and community members. However, the limited number of accounts in the available data may reflect lack of inquiry or reporting of such cases. In contrast, the review clearly demonstrated that mobile-based programs may inadvertently reinforce the digital divide, particularly in terms of access to information. The technology also has the potential to reflect the existing gendered context, rather than enabling a new dynamic, with little to no influence on gender relations. In some cases, it was unclear if changes in men’s engagement translated into positive and equitable outcomes for women, or represented new means for men to expand control over women. Other instances of limited influences on gender relations were women’s dependency on men’s approval or assistance to use mobile devices. Some unfavorable changes were also described such as tensions between couples regarding who ultimately owned the mobile phone and how it would be used.

This points to an important recognition that while mHealth interventions can significantly improve women’s health and well-being, the expectation that mobile health programs will drastically alter gender inequities within relationships deserves some scrutiny. Unless there is a conscientious effort to design mHealth initiatives that promote equitable relationships between men and women, the scale-up of some current programs may prove harmful to women. At the same time, the literature on the influence of mHealth interventions on gender relations is relatively weak. While we identified positive and negative outcomes, to some extent the evidence remains inconclusive. Some selected studies reported that determining the full extent of whether women’s involvement in the intervention impacted gender structures was beyond the scope of study, and only two articles purposed from the outset of the evaluation to examine gender-based dynamics. In addition, the majority of studies used a single group post-test design which was unable to differentiate between direct effects of the intervention, such as autonomy or empowerment, on gender relations versus the added value of mobile technology itself. Thus, when coupled with the overall insufficient quality of the evidence base, we found that there was a scarcity of research purposefully investigating this topic. Rigorous research that examines gender relations either as a stand-alone study or embedded within existing experimental mHealth trials is urgently needed.

Nonetheless, the commonality of findings across various settings can assist in the development of appropriately empowering and gender-transformative mobile health programs. Experience has shown that current gender inequalities have weakened attempts to scale-up health and development interventions [43]. This review demonstrates that more careful planning and investigation of potential gender implications prior to implementation would likely be informative when coupled with rigorous program evaluation methodologies. Although mhealth programs are often considered inherently beneficial, we recommend that future efforts assess the transformative and potentially exploitative effects of mobile initiatives. Such is needed not only from the perspective of women, but men and community members as well.

### Limitations

The findings of this review were subject to limitations. Our analysis did not apply a tiered system to synthesize results by rigor category given the small number of identified studies. In addition, the quality ratings often reflected the methodological designs adopted for the primary outcome of interest, rather than peripheral findings related to gender relations. Given the role that socio-cultural factors play in defining relations between men and women, the interpretation of findings is also highly context-specific and may not be generalizable to other settings. Finally, it is possible that some studies were overlooked given that the search was limited to articles published in English and those which were available online. As a result, the findings do not represent experiences drawn from unpublished and non-English texts. However, among electronically accessible English-language studies, we applied a considerably thorough and robust search strategy.

## Conclusion

The scarcity and low quality of the literature on the effect of mHealth interventions on gender relations urgently necessitates more rigorous research. Current findings suggest that mobile interventions can beneficially influence gender relations, while at the same time strain and reinforce existing power imbalances. Given the increasing number of health and development interventions delivered by mobile technologies, more care should be taken to implement and evaluate mHealth program that promote, rather than hinder gender equality.

## Abbreviations

AIDS: Acquired immune deficiency syndrome; HIV: Human immunodeficiency virus; SMS: Short message service.

## Competing interests

The authors declare that they have no competing interests.

## Authors’ contributions

LJ designed the scope of the review and the search strategy. LJ and LG jointly conducted the search, screened citations, read and appraised the literature, and summarized findings. LJ led the content analysis and wrote the first draft of the manuscript. LG edited subsequent drafts and constructed the figures and tables. LJ and LG jointly revised the present manuscript version. Both authors read and approved the final manuscript.
